# *Adenophora divaricata* Franch. & Sav. Attenuates Particulate Matter-Induced Inflammatory Responses in RAW264.7 Macrophage Cells

**DOI:** 10.3390/cells15080666

**Published:** 2026-04-09

**Authors:** Ji-Hye Ha, Ba-Wool Lee, Da-Hye Yi, Seong-Hun Jeong, Ju-Hong Kim, Hyeon Jin Lee, Yun-Hye Kim, Ju Hwan Jeong, Hyun-Jae Jang, Woo Sik Kim, Ji-Young Park, Hyung Jae Jeong, Hyung-Jun Kwon, Tae-Won Kim, Je-Won Ko, In-Chul Lee

**Affiliations:** 1 Functional Biomaterial Research Center, Korea Research Institute of Bioscience and Biotechnology, Jeongeup-si 56212, Jeollabuk-do, Republic of Korea; jihye2640@kribb.re.kr (J.-H.H.); paul0706@kribb.re.kr (B.-W.L.); ydh1013@kribb.re.kr (D.-H.Y.); jsh0830@kribb.re.kr (S.-H.J.); wnghd93@kribb.re.kr (J.-H.K.); tgb04261@kribb.re.kr (H.J.L.); ysun0021@kribb.re.kr (Y.-H.K.); juhwanjeong@kribb.re.kr (J.H.J.); water815@kribb.re.kr (H.-J.J.); kws6144@kribb.re.kr (W.S.K.); loveme@kribb.re.kr (J.-Y.P.); hjeong21@kribb.re.kr (H.J.J.); hjkwon@kribb.re.kr (H.-J.K.); 2 College of Veterinary Medicine, Chungnam National University, Daejeon 34131, Republic of Korea; taewonkim@cnu.ac.kr; 3 College of Veterinary Medicine, Chonnam National University, Gwangju 61186, Republic of Korea

**Keywords:** particulate matter, pulmonary inflammation, TXNIP, NLRP3 inflammasome

## Abstract

**Highlights:**

**What are the main findings?**

- Particulate matter (PM) triggers pulmonary inflammation through activation of the TXNIP/NLRP3 inflammation pathway, although particular mechanisms remain unclear.
- This study investigates the anti-inflammatory potential of *Adenophora divaricata* Franch. & Sav. (AD) and its regulatory effects on TXNIP/NLRP3 inflammasome signaling in PM-stimulated in RAW264.7 cell and PM-induced lung inflammation mice.

**What are the implications of the main findings?**

- AD significantly attenuated inflammatory responses in PM-stimulated in RAW264.7 cell and PM-induced lung inflammation mice. Treatment reduced inflammatory cell infiltration and pro-inflammatory cytokine levels in bronchoalveolar lavage fluid.
- AD markedly suppressed ROS-mediated TXNIP/NLRP3 inflammasome activation, identifying TXNIP as a critical regulatory target. Histological analysis showed improved lung tissue integrity, including reduced inflammatory cell infiltration and reduced alveolar wall thickening.
- Findings suggest AD as a promising therapeutic candidate for PM-induced pulmonary inflammation.

**Abstract:**

Particulate matter (PM) is a complex mixture of airborne solid particles and liquid droplets originating from various environmental sources, and it has been implicated in the initiation, development, and progression of pulmonary inflammation and respiratory diseases. However, the underlying associated molecular mechanisms remain unclear. *Adenophora divaricate* Franch. & Sav. (AD) is a medicinal herb classified within the Campanulaceae family and genus *Adenophora*, with a broad geographic distribution across East Asia, including Korea, Asia, and Russia. In this study, we investigated the mechanisms underlying the effects of AD on PM-induced lung inflammation in both PM-stimulated RAW264.7 cells and PM-exposed mice. Considering that the reactive oxygen species (ROS)-mediated thioredoxin-interacting protein (TXNIP) and NOD-like receptor pyrin domain containing (NLRP3) inflammasome pathway plays a role in PM-induced inflammatory responses, we focused on determining whether AD exerts its anti-inflammatory effects through modulation of this signaling pathway. The anti-inflammatory properties of the methanolic extract of AD were evaluated using PM-stimulated RAW264.7 cells and PM-exposed mice. PM was administered intranasally to mice for 7 days, whereas AD or dexamethasone was orally administered for the same duration. AD treatment significantly attenuated pulmonary inflammation, as evidenced by reduced inflammatory cell counts and decreased cytokine levels in bronchoalveolar lavage fluid. In addition, AD decreased oxidative stress marker (ROS and thiobarbituric acid reactive substances) while increasing glutathione content, leading to suppression of TXNIP/NLRP3 inflammasome expression. Histopathological analysis revealed a marked alleviation of inflammatory responses in lung tissue, characterized by diminished inflammatory cell infiltration and reduced alveolar wall thickening. Collectively, these findings suggest ROS-mediated TXNIP serves as a key regulatory factor, and AD may serve as a potential therapeutic agent for pulmonary inflammation.

## 1. Introduction

Recently, rapid industrialization has led to a significant increase in ambient particulate matter (PM) concentrations, which has emerged as a critical global public health concern. PM is widely associated with various health issues, including respiratory diseases, diabetes, cancer, and cardiovascular disorders [1,2]. PM, a major component of air pollution, is composed of fine liquid droplets and solid particles primarily derived for gaseous emissions and vehicle various inflammatory conditions [3,4]. Prior research has shown that PM exposure can trigger oxidative stress and inflammatory reactions. This process promotes the activation of recruitment of inflammatory cells and enhances the generation of reactive oxygen species (ROS). In addition, PM exposure is associated with elevated secretion of pro-inflammatory mediators such as tumor necrosis factor-α (TNF-α) and interleukin-6 (IL-6) [5,6]. Despite increasing evidence of PM-induced pulmonary inflammation, the precise molecular mechanism has not been adequately elucidated, and there are currently no effective pharmacological approaches to manage the condition [7]. Therefore, it is crucial to explore and identify promising therapeutic compounds and establish innovative strategies to alleviate PM-triggered lung inflammation.

ROS-induced oxidative stress contributes to the development of acute lung injury (ALI) by activating the TXNIP/NLRP3 inflammasome and nuclear factor (NF)-κB signaling pathway [8]. Thioredoxin-interacting protein (TXNIP) is a stress inducible regulator of cellular redox homeostasis that suppresses the anti-oxidant activity of thioredoxin (TRX) [9]. During oxidative stress, TXNIP separates from TRX and subsequently binds to the NOD-like receptor pyrin domain containing (NLRP3) inflammasome. This interaction promotes activation of caspase-1 and facilitates the processing of pro-inflammatory cytokines, including interleukin-1β, into their mature forms [10,11]. Exposure to PM, a major environmental risk factor for respiratory diseases, induces excessive generation of ROS in airway epithelial cells and alveolar macrophages, thereby upregulating TXNIP expression and promoting NLRP3 inflammasome activation [12]. In addition, the TXNIP/NLRP3-mediated inflammatory cascade has been implicated in the pathogenesis of acute respiratory distress syndrome, and chronic inflammatory pulmonary diseases following dust [13]. Collectively, the TXIP/NLRP3 inflammasome is a critical molecular link between PM-induced oxidative stress and pulmonary inflammation, highlighting TXNIP as a potential therapeutic target for environment associated lung diseases.

*Adenophora divaricate* Franch. & Sav. (AD), a member of the family Campanulaceae and genus *Adenophora*, is commonly distributed in the Republic of Korea, China, Japan, and Russia [14]. It has been reported that ethanol extracts of *Adenophora* species exhibit anti-tumor activity, whereas the ethyl acetate fraction derived from the roots displays anti-oxidant properties and alleviates oxidative stress and inflammatory responses in high-fat diet-induced obese mouse models, accompanied by improvements in insulin resistance [15,16,17]. Consistent with these findings, *A. triphylla* var. japonica has been reported to contain diverse bioactive constituents, including saponins, flavonoids, and polysaccharides, which have anti-inflammatory, anti-oxidant, and immunomodulatory activities [18,19]. Extracts of bioactive compounds derived from *Adenophora* species suppress the phosphorylation of MAPKs, thereby attenuating downstream NF-κB activation and reducing the expression of pro-inflammatory cytokines such as TNF-α, IL-6, and IL-1β [18,20]. Furthermore, previous studies have suggested that these inhibitory effects on the MAPK/NF-κB signaling pathways indicate that *Adenophora* may exert its anti-inflammatory activity by regulating key intracellular signaling pathway involved in immune responses and inflammatory processes [18,20,21]. Despite accumulating evidence supporting the biological activities of *Adenophora* species, the role of AD in modulating lung inflammation and respiratory disorders has received limited investigation, suggesting its investigation as a potential therapeutic candidate for respiratory diseases.

## 2. Materials and Methods

### 2.1. UPLC Q-ToF/MS Analysis

LC-QTOF/MS analysis was performed using a Shimadzu Nexera XS UPLC system (Shimadzu, Kyoto, Japan) coupled with a SCIEX X500R quadrupole time-of-flight mass spectrometer (SCIEX, Framingham, MA, USA). Chromatographic separation was achieved on an ACCQUITY UPLC C18 column (2.1 × 100 mm, 1.7 µm; Waters, Milford, MA, USA) maintained at 35 °C.

The mobile phases consisted of solvent A (0.1% formic acid in water) and solvent B (0.1% formic acid in acetonitrile). The gradient elution program was as follows: 0 min, 95%, 75% A; 20 min, 50% A; 28 min, 10% A; 35 min, 10% A; and 45 min, and 95% A. The flow rate was wet at 0.3 mL/min, and the injection volume was 3 µL.

Mass spectrometric analysis was conducted in both negative and positive electrospray ionization (ESI) modes using information-dependent acquisition (IDA). The ion spray voltage was wet to 5500 V in positive mode and −4500 V in negative mode. The source temperature was 500 °C. Ion source gases (GS1 and GS2) were set to 50 psi, and the curtain gas was set to 30 psi.

TOF MS and MS/MS data were acquired over the m/z ranges of 100–1500 and 50–1500, respectively. The declustering potential (DP) was set to 80 V in positive mode −80 V in negative mode. The collision energy (CE) was set to 35 ± 15 V in positive mode, and −35 ± 15 V in negative mode. High mass accuracy (within ±2 ppm) was achieved using external calibration.

### 2.2. Chemicals and Materials

PM (≤10 μm, 95%, ISO-12103-1; *Road Vehicles–Test Dust for Filter Evaluation*; International Organization for Standardization: Geneva, Swizerland, 2016) was purchased from Powder Technology Industry Inc. According to our previous study, this PM primarily consists of silicon (50.71%), aluminum (48.48%), and iron (7.25%), reflecting the typical mineral composition of PM [22]. AD was obtained from the Korea Plant Extract Bank (LPM; Korea Research Institute of Bioscience and Biotechnology) from aerial parts of *Adenophora* species using methanol as the extraction solvent, and the sample used in this study corresponds to voucher/sample number KPM025-023. TXNIP-specific siRNA (catalog no. 4390771), together with a scrambled negative control siRNA (catalog no. 4390843), were also obtained from Ambion (Waltham, MA, USA). The NLRP3 inhibitor MCC950 and dexamethasone (DEX) were acquired from Sigma-Aldrich (St. Louis, MO, USA), Lipofectamine™ RNAiMAX transfection reagent was supplied by Invitrogen (Waltham, MA, USA). ROS detection assay kit (DCF-DA; CellRox^®^ green reagent; Thermo Scientific, Waltham MA, USA), EZ-Glutathione Assay Kit and EZ-Lipid Peroxidation (TBARS) Assay Kit (DoGen, Seoul, Republic of Korea) were used.

### 2.3. Cell Culture and Cell Viability Assay

Murine macrophage RAW264.7 cells were maintained in DMEM media (Gibco, San Diego, CA, USA) supplemented with 10% FBS and 1% antibiotics (Gibco). Cell viability was determined using a WST-1 assay. Cells were seeded in 96-well plates at a density 5 × 10^4^ cells per well and incubated for 24 h prior to treatment with AD (0–200 μg/mL). Absorbance was recorded at 450 nm using a microplate reader (iMark™, Bio-Rad Laboratories, Richmond, CA, USA), and viability was calculated relative to untreated control cells, which were defined as 100%.

### 2.4. Measurement of Inflammatory Cytokines and Oxidative Stress Markers

RAW26.7 cells were pretreated with AD at (50–200 μg/mL) or DEX (10 μg/mL) for 1 h followed by stimulation with PM (200 μg/mL). After 24 h of incubation, culture supernatants were harvested and the concentrations of TNF-α IL-1β, and IL-6 were quantified using ELISA kits (BD Bioscience, San Jose, CA, USA) according to the supplier’s guidelines. ROS production was assessed using the CellRox^®^ Green Reagent (Thermo Scientific), respectively, following the instructions provided by the manufacturers. Additionally, the content of recued glutathione (GSH) and thiobarbituric acid reactive substances (TBARS) was assessed by using EX-Glutathione assay kit and EX-lipid peroxidation (TBARS) assay kit (DoGen) according to the provided instructions. Optical density was recorded at 450 nm using a microplate reader (Bio-Rad Laboratories).

### 2.5. Immunoblotting

Cells lysates were prepared with M-PER™ reagent containing protease and phosphatase inhibitor cocktail (Thermo Fisher Scientific). Lung tissues were homogenized in T-PER reagent supplemented with the same inhibitor cocktail (Thermo Fisher Scientific). Protein expression associated with the signaling pathway was examined by immunoblotting following a previously reported protocol [22]. Primary antibodies and dilutions: pro-caspase-1, and pro-IL-1β (1:1000 dilution, Abcam, Cambridge, UK), TXNIP and NLRP3 (1:1000 dilution, Novus Bio, Centennial, CO, USA), β-actin (1:1000 dilution, Cell Signaling Technology, Danvers, MA, USA). Densitometric analysis for each protein band was performed using chemiluminescent scanner (LI-COR, Biosciences, Lincoln, NE, USA).

### 2.6. Small Interfering RNA (siRNA) Transfection

RAW264.7 cells were transfected with TXNIP-specific siRNA and scramble control siRNA (Ambion) using Lipofectamine™ RNAiMAX reagent (Invitrogen) following the supplier’s forward transfection procedure. Each siRNA was used at a final concentration of 20 nM. After TXNIP suppression, cells were pretreated to AD (200 μg/mL) for 1 h and subsequently stimulated with PM (200 μg/mL). Following a 2 h incubation period, cells were harvested to examine activation of the TXNIP/NLRP3 inflammasome signaling pathway by immunoblotting and immunofluorescence (IFA).

### 2.7. NLRP3 Inhibitor

RAW264.7 cells were pretreated with an NLRP3 inhibitor and AD (200 μg/mL) for 1 h, followed by treatment with PM at a concentration of 200 μg/mL. After a 2 h incubation, the cells were collected for analysis and the levels of TXNIP/NLRP3 inflammasome pathway was evaluated using immunoblotting and IFA. NLRP3 inhibitor according to the manufacturer’s protocol, with each NLRP3 inhibitor applied at a final concentration of 5 μM.

### 2.8. Immunofluorescence and Microscopy

RAW264.7 cells were incubated with the primary antibody for 1 h at 37 °C, followed by incubation with the corresponding secondary antibodies for an additional hour at 37 °C. The antibodies used were as follows: TXNIP and NLRP3 (1:200 dilution; Novus Bio), anti-Mouse IgG (whole molecule)-TRITC antibody (1:100 dilution; Sigma-Aldrich), and anti-Rabbit IgG (whole molecule)-FITC antibody (1:1000 dilution; Thermo Fisher Scientific). After staining, the cells were mounted on glass slides using ProLong™ Gold Antifade Reagent containing DAPI (Invitrogen). Fluorescence images were captured using a Zeiss fluorescence microscope (Zeiss, Jena, Germany) equipped with a Zeiss objective lens, and the fluorescence intensity was quantified with Zeiss Zen 3.11 software (Zeiss Microsystems).

### 2.9. Animal Experimental Procedure

All animal procedures were approved by Institutional Animal Care and Use Committee of the Korea Research Institute of Bioscience and Biotechnology (KRIBB) (KRIBB-AEC-24280; Approval Date: 25 February 2025). Mice were allocated into five groups (*n* = 7/group): normal control (NC), PM treatment (PM, 5 mg/kg), DEX treatment (3 mg/kg), and AD-treated groups (AD, 50 mg/kg and 100 mg/kg). PM was suspended in sterile phosphate-buffered saline (PBS, 7.4) and sonicated for 1 min before administration. One hour after oral administration of DEX or AD, mice in the PM and AD treatment groups received 50 μL of the PM suspension intranasally once daily for 7 consecutive days under anesthesia with 2–3% isoflurane inhalation. Dose levels for DEX and AD doses were selected based on the previous in vivo studies demonstrating effective anti-inflammatory responses in mice within similar dosing ranges [23,24]. Treatments were administered daily for 7 consecutive days. After the final administration, mouse was anesthetized, and bronchoalveolar lavage fluid (BALF) was obtained following an established protocol [25]. BALF samples were centrifuged (800 rpm, 5 min, 4 °C); the supernatant was used for cytokine analysis, whereas the cell pellet was resuspended in PBS for inflammatory cell enumeration using an Auto Hematology Analyzer Mindray Animal Care, BC-5000 Vet (Shenzhen, China). The levels of TNF-α and IL-6 in the BALF were measured by ELISA kits (BD Bioscience), as described above.

### 2.10. Histopathological Analysis

Lung tissue was preserved in 10% neutral-buffered formalin. Tissues preparation was performed as previously described [23]. The samples were paraffin embedded and sectioned at a thickness of 4 μm. For histological assessment of airway inflammation, lung tissue was stained with hematoxylin and eosin (H&E; BBC Biochemical, Mount Vemon, WA, USA). Microscopic examination was performed using a Leica optical microscope scanner equipped with a 10× and 20× and objective lens. The main histological lesions, alveolar wall thickening and inflammatory infiltration, were graded as follow: 0, no lesions; 1, mild; 2, moderate; and 3, severe [24].

### 2.11. Statistical Analysis

All results are presented as mean ± SD. Statistical evaluations were conducted using GraphPad Prism 5 (GraphPad 5 Software, San Diego, CA, USA). Group differences were assessed by one-way ANOVA followed by Tukey’s post hoc test, and values of *p* ≤ 0.05 were regarded as statistically significant.

## 3. Results

### 3.1. Chemical Constituents of AD

**Figure 1:**
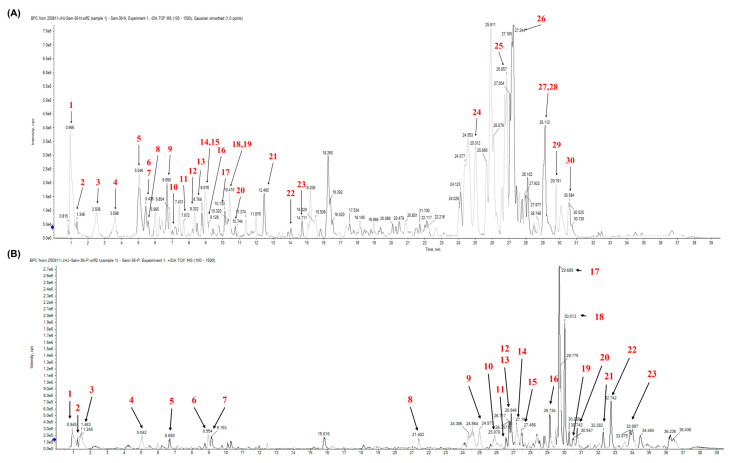
Representative chromatography profiles of the ethyl acetate fraction obtained from AD analyzed by ultra-performance liquid chromatography coupled with quadrupole time-of-flight mass spectrometry (UPLC–Q–TOF–MS). The negative (**A**) and positive (**B**) ionization modes.

UPLC-Q-TOF-MS was conducted to characterize the chemical composition of AD in both negative and positive ionization modes. A total of 30 metabolites were detected in the negative-ion mode, mainly comprising flavonoids (*n* = 10), phenolic acids (*n* = 6), and fatty acids (*n* = 6). In the positive-ion mode, the identified constituents included amino acids (*n* = 3), phenolic acids (*n* = 3), flavonoids (*n* = 2), an alkaloid (*n* = 1), fatty acids (*n* = 5), and phospholipids (*n* = 2). Structural assignment was achieved through interpretation of MS/MS fragmentation pattern, comparison with reference standards, and literature verification (Figure 1 and Table 1 and Table 2).

### 3.2. Effects of AD on Cell Viability and Pro-Inflammatory Cytokines in PM-Stimulated RAW264.7 Cells

**Figure 2:**
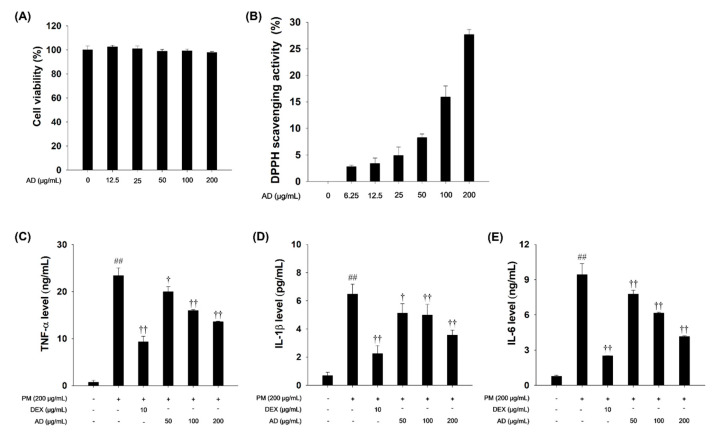
Effect of AD on inflammatory responses in PM-stimulated RAW264.7 cells. (**A**) Cell viability after 24 h incubation following AD treatment. (**B**) DPPH scavenging activity was assessed in a concentration-dependent manner of AD treatment (6.25–200 μg/mL). (**C**–**E**) TNF-α IL-1β, and IL-6 levels determined using ELISA, respectively. Data are presented as means ± SD (*n* = 3). Significant: ^##^ compared with the NC group (*p* < 0.05); ^†^, ^††^ compared with PM-stimulated cells (*p* < 0.05 and *p* < 0.01).

AD was not cytotoxic to RAW264.7 cells at concentrations of 0, 12.5, 50, 100, and 200 μg/mL (Figure 2A). PM-stimulated cells showed markedly elevated pro-inflammatory cytokine levels (TNF-α, IL-1β, and IL-6) compared to that of non-stimulated cells. However, these levels decreased in dose-dependent manner following treatment with AD (Figure 2B,C).

### 3.3. Effects of AD on the Oxidative Stress and TXNIP/NLRP3 Inflammasome Pathway in PM-Stimulated RAW264.7 Cells

**Figure 3:**
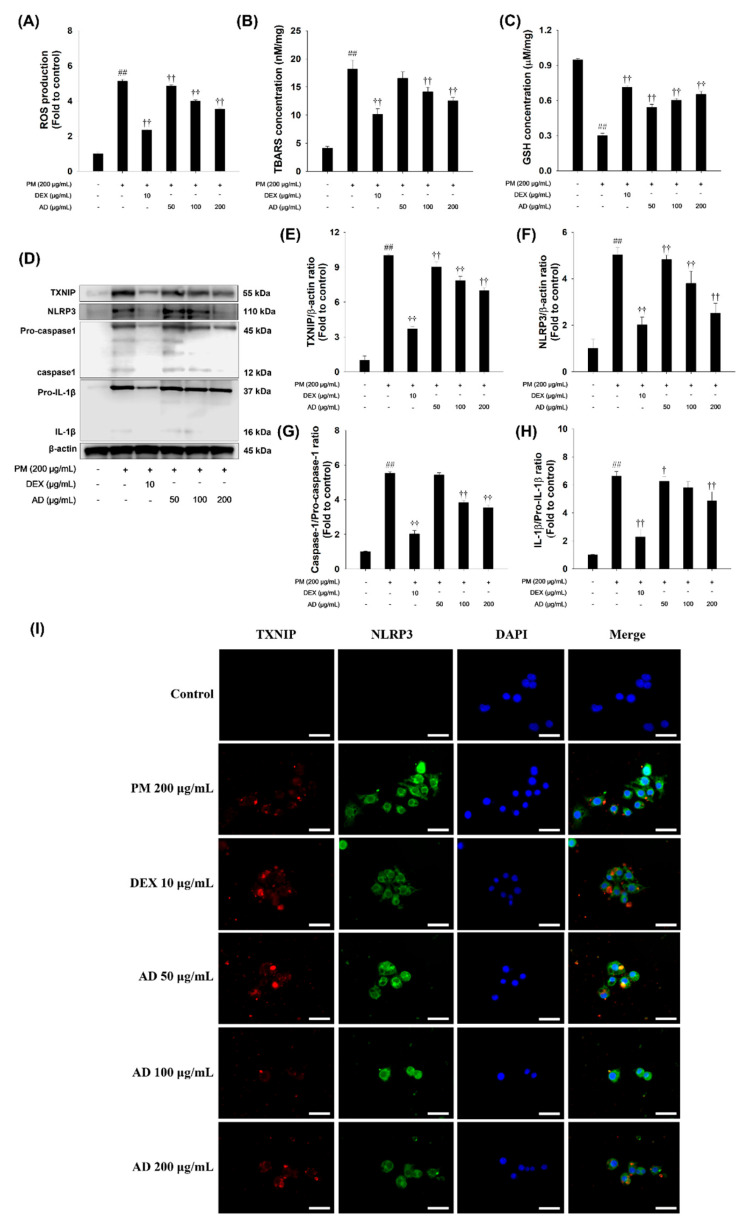
Effect of AD on TXNIP/NLRP3 inflammasome activation in PM-treated RAW264.7 cells. Oxidative stress parameters were evaluated, including (**A**) ROS production, (**B**) TBAR levels and (**C**) GSH content. (**D**) Western blot images showing protein expression levels. (**E**–**H**) Quantitative densitometric analysis of the indicated protein. (**I**) Representative double-IFA staining images illustrating TXNIP and NLRP3 expression in cells stimulated with PM (200 μg/mL), DEX (10 μg/mL), and AD (50, 100, and 200 μg/mL). Data are presented as means ± SD (*n* = 3). Significant: ^##^ compared with the NC group (*p* < 0.05); ^†^, ^††^ compared with PM-stimulated cells (*p* < 0.05 and *p* < 0.01).

We investigated the effects of AD on oxidative stress and TXNIP/NLRP3 inflammasome in PM-stimulated RAW264.7 cells (Figure 3). PM stimulation significantly increased oxidative stress markers, including ROS and TBARS, while markedly decreasing GSH content (Figure 3A–C). In addition, PM-stimulated cells exhibited upregulation of TXNIP and NLRP3, along with increased levels of the activation forms of caspase-1 and IL-1β, compared to that of non-stimulated cells. In contrast, AD treatment significantly reduced ROS and TBARS levels, enhancing GSH content. Furthermore, AD dose-dependently suppressed the upregulation of TXNIP and NLRP3, as well as the activation of caspase-1 and IL-1β in PM-stimulated RAW264.7 cells (Figure 3D–H). Additionally, double-IFA staining revealed marked accumulation of TXNIP and NLRP3 in PM-stimulated cells compared with untreated cells. AD treatment markedly attenuated the fluorescence intensity of both TXNIP and NLRP3. Merged images further demonstrated that AD significantly reduced the co-localization of TXNIP and NLRP3, suggesting that AD suppresses PM-induced activation of the NLRP3 inflammasome in macrophages (Figure 3I).

### 3.4. Effects of TXNIP-Specific siRNA on the TXNIP/NLRP3 Inflammasome Signaling Pathway in PM-Stimulated RAW264.7 Cells

**Figure 4:**
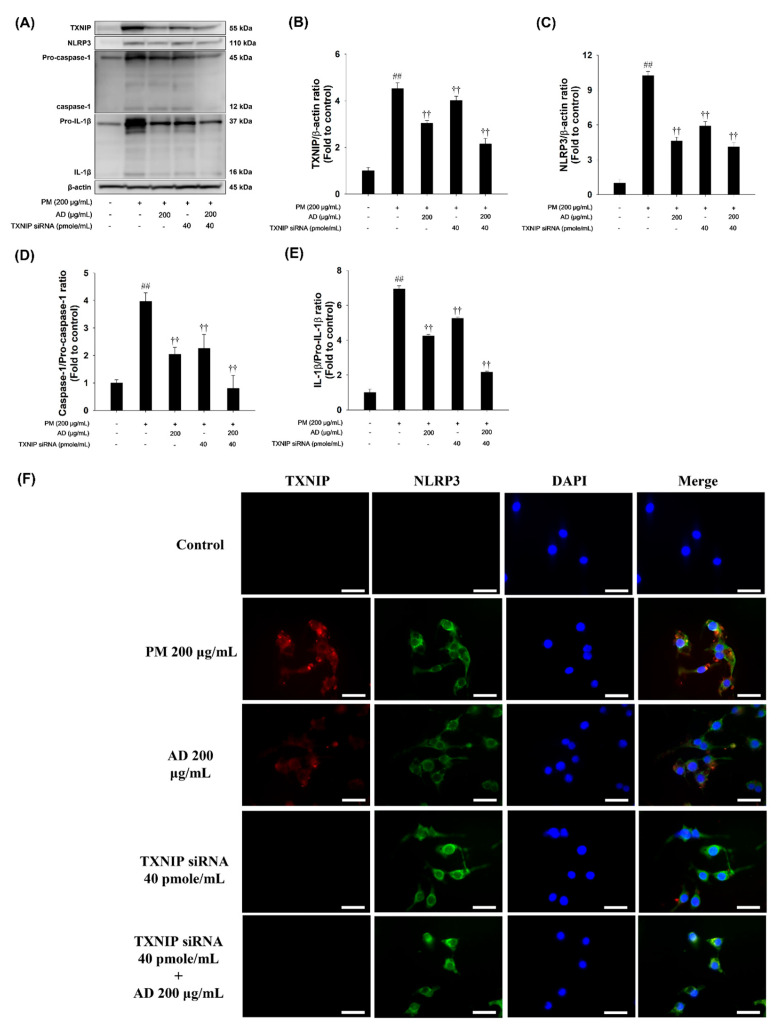
Effect of TXNIP knockdown and AD treatment on the TXNIP/NLRP3 inflammasome pathway in PM-stimulated RAW264.7 cells. (**A**) Western blot images showing protein expression levels. (**B**–**E**) Densitometric quantification of the indicated proteins. (**F**) IFA images showing TXNIP and NLRP3 expression in cells with siRNA mediated TXNIP knockdown. Scale bar = 20 μm. Data are presented as means ± SD (*n* = 3). Significant: ^##^ compared with the NC group (*p* < 0.05); ^††^ compared with PM-stimulated cells (*p* < 0.05 and *p* < 0.01).

To examine the role of TXNIP in PM-stimulated inflammatory, RAW264.7 cells were transfected with TXNIP siRNA and then treated with PM (200 μg/mL). As shown in Figure 4A–E, TXNIP knockdown decreased NLRP3 expression and reduced the activated forms of caspase-1 and IL-1β, compared with the PM-only group. Double-IFA staining further showed that PM exposure increased fluorescence signals and cytoplasmic co-localization of TXNIP and NLRP3, indicating inflammasome activation. In contrast, TXNIP-specific siRNA reduced both fluorescence intensity and their co-localization (Figure 4F). Therefore, TXNIP silencing attenuates inflammasome activation and supports the anti-inflammatory effect of AD observed in earlier experiments.

### 3.5. Effects of an NLRP3 Inhibitor on TXNIP/NLRP3 Inflammasome Signaling in PM-Stimulated RAW264.7 Cells

**Figure 5:**
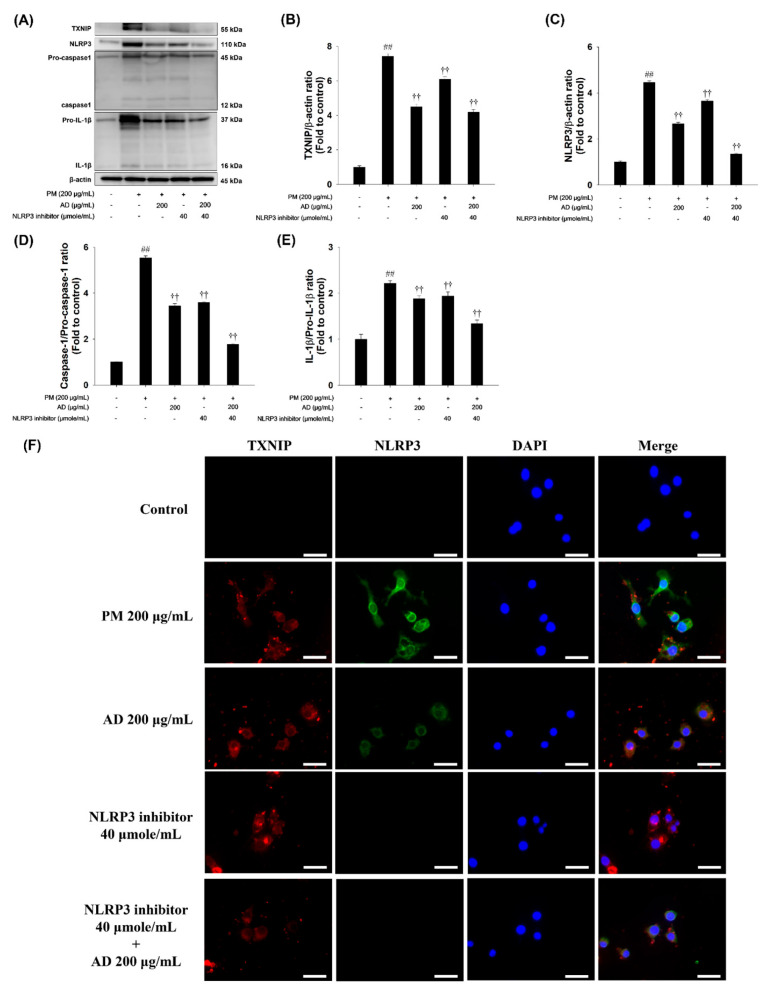
Effect of NLRP3 inhibition and AD on the TXNIP/NLRP3 inflammasome signaling pathway in PM-stimulated RAW264.7 cells. (**A**) Protein expression detected on the Western blot gels. (**B**–**E**) Relative densitometric values of each protein. (**F**) IFA for TXNIP and NLRP3 in inhibitor mediated NLRP3 inhibition cells. Scale bar = 20 μm. Data are presented as means ± SD (*n* = 3). Significant: ^##^ compared with the NC group (*p* < 0.05); ^††^ compared with PM-stimulated cells (*p* < 0.05 and *p* < 0.01).

To evaluate the inhibitory effect of NLRP3 suppression on PM-induced inflammasome activation, RAW264.7 cells were treated with PM (200 μg/mL) in the presence or absence of an NLRP3 inhibitor. Co-treatment with the NLRP3 inhibitor markedly reduced the PM-induced expression of TXNIP and NLRP3, as well as the levels of active caspase-1 and IL-1β. Double-IFA staining for TXNIP and NLRP3 further demonstrated that PM exposure significantly enhanced the fluorescence intensity and cytoplasmic co-localization of the two proteins, indicating inflammasome activation. In contrast, treatment with the NLR3 inhibitor greatly diminished both the fluorescence intensity and cytoplasmic co-localization of TXNIP and NLRP3 (Figure 5E). Collectively, NLRP3 inhibition alleviates inflammasome activation, thereby providing additional evidence to support the anti-inflammatory mechanism of AD observed in previous experiments.

### 3.6. Effects of AD on Inflammatory Cell Counts and Pro-Inflammatory Cytokines in BALF

**Figure 6:**
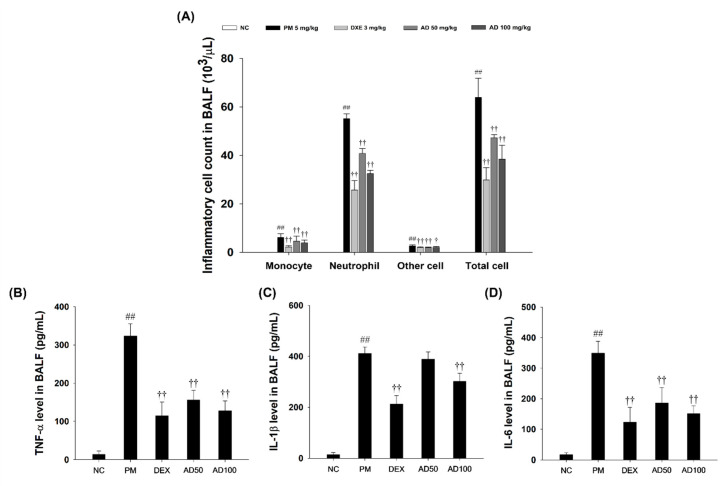
Effect of AD on pathophysiological changes observed in BALF. (**A**) Differential counts of inflammatory cells counted in a double-blind manner on 3 areas for each slide. The levels of (**B**) TNF-α, (**C**) IL-1β, and (**D**) IL-6 in BALF. Data are presented as means ± SD (*n* = 7). Significant: ^##^ compared with NC group (*p* < 0.05); ^†^, ^††^ compared with PM-induced group (*p* < 0.05 and *p* < 0.01).

Mice exposed to PM exhibited a marked increase in inflammatory cell counts compared to those in the normal control group (Figure 6A). Conversely, treatment with AD notably mitigated PM-induced infiltration of inflammatory cells. Moreover, TNF-α, IL-1β, and IL-6 levels were considerably higher in PM-exposed mice than in normal control group. In contrast, administration of AD led to a dose-dependent reduction in Th-1 type cytokine expression relative to that in the PM-treated group (Figure 6B,C).

### 3.7. Effects of AD on LPS-Induced Lung Histological Changes in Lung Tissue

**Figure 7:**
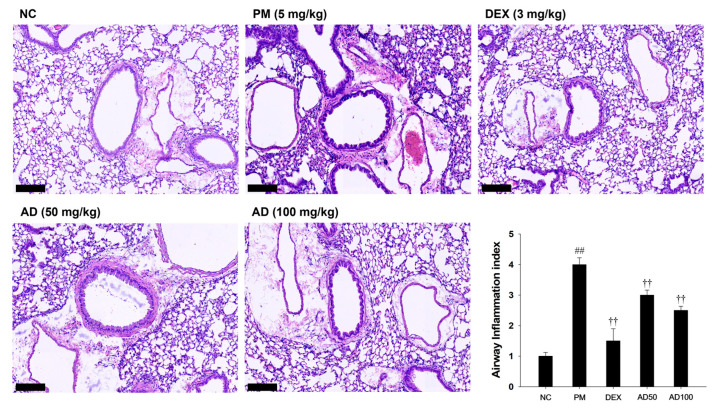
Effects of AD on airway inflammation in the lung of PM-induced mice. Representative histological images of lung sections stained with hematoxylin and eosin are shown (scale bar = 100 μm). Quantitative analysis of the airway inflammation, respectively. Data are presented as means ± SD (*n* = 7). Significant: ^##^ compared with NC group (*p* < 0.05); ^††^ compared with PM-induced group (*p* < 0.05 and *p* < 0.01).

The effect of AD on PM-induced histopathological alterations in lung tissues was evaluated (Figure 7). In the lung sections of PM-exposed mice, significant thickening of the alveolar walls and increased infiltration of inflammatory cells were observed compared with those in the normal control group. In contrast, AD administration alleviated both alveolar wall thickening and inflammatory cell infiltration, leading to a marked reduction in lesion scores relative to those in the PM-exposed mice.

### 3.8. Effects of AD on Oxidative Stress and TXNIP/NLRP3 Inflammasome Pathways in Lung Tissue

**Figure 8:**
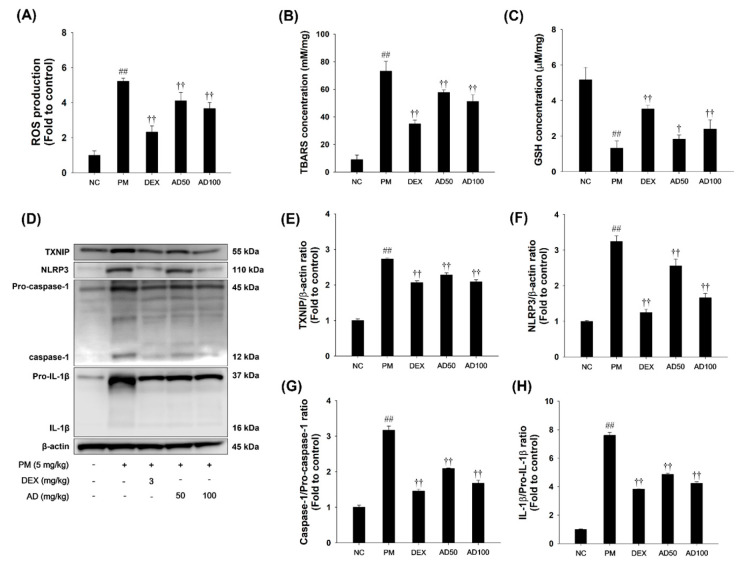
Effect of AD on the activation of the TXNIP/NLRP3 inflammasome in PM-induced mice. Oxidative stress indicator, including (**A**) ROS production, (**B**) TBAR levels and (**C**) GSH content. (**D**) Western blot images showing protein expression levels. (**E**) TXNIP, (**F**) NLRP3, (**G**) caspase-1, and (**H**) IL-1β. Data are presented as means ± SD (*n* = 7). Significant: ^##^ compared with NC group (*p* < 0.05); ^†^, ^††^ compared with PM-induced group (*p* < 0.05 and *p* < 0.01).

To elucidate the mechanism by which AD alleviates inflammation in PM-exposed mice, we examined its effect on the oxidative stress and TXNIP/NLRP3 inflammasome pathway (Figure 8). PM-exposed mice exhibited significantly increased ROS and TBARS levels, along with decreased GSH content. In addition, TXNIP and NLRP3 were markedly upregulated, accompanied by increased levels of activated forms of caspase-1 and IL-1β in lung tissues. In contrast, AD treatment significantly reduced ROS and TBARS levels while increasing GSH content (Figure 8A–C). Furthermore, AD treatment significantly downregulated TXNIP and NLRP3 expression and suppressed the activation of caspase-1 and IL-1β (Figure 8D–G).

## 4. Discussion

Accelerated industrialization has led to increased PM in the atmosphere globally, raising concerns about respiratory health because of its primary inhalation pathway through the respiratory system [13,26]. As fine particles primarily enter the body through the respiratory tract, their elevated levels may pose significant risks to respiratory function and initiate the development of diseases [4,27]. Despite various diseases associated with PM exposure, the fundamental pathophysiological processes involved remain unclear. The present study elucidated the possible protective mechanisms against PM-induced toxicity, in turn introducing potential therapeutic approaches that may effectively alleviate its adverse health effects. The present study demonstrated that AD effectively attenuates PM-induced airway inflammation in PM-stimulated RAW264.7 cell and exposed mice. Exposure to PM led to higher numbers of inflammatory cells and increased cytokine concentrations in BALF, along with noticeable accumulation of inflammatory cells infiltration of the alveoli and bronchi. Consistently, AD treatment significantly reduced cytokine level and inflammatory cell accumulation, suggesting a direct anti-inflammatory effect against PM-induced airway inflammation. Furthermore, the attenuation of airway inflammation following treatment with AD in PM-stimulated RAW264.7 cells, and PM-exposed mice was associated with decreased TXNIP expression, which may have subsequently inhibited the activation of the NLRP3 inflammasome. The involvement of TXNIP in AD treatment mediated regulation of airway inflammation was further confirmed using TXNIP siRNA-treated RAW264.7 cells.

Exposure to PM induces airway inflammatory responses characterized by the excessive generation of ROS and dysregulated production of pro-inflammatory cytokines (IL-6, IL-1β, and TNF-α) [4]. TXNIP, an endogenous inhibitor of thioredoxin (TRX), has been implicated in the progression of various pathological conditions, including myocardial injury, pulmonary disease, and multiple inflammatory diseases [28]. Under oxidative stress, TXNIP dissociates from oxidized TRX and subsequently associates with the NLRP3 inflammasome [29,30]. This interaction promotes NLRP3 inflammasome activation, leading to caspase-1 mediated cleavage of IL-1β and IL-18 into their mature, bioactive forms, thereby exacerbating inflammatory responses [31]. Additionally, the enhanced activation of TXNIP-mediated inflammatory signaling is reflected in distinct histopathological alterations in the lungs [13,32]. In particular, increased TXNIP activity is accompanied by pronounced accumulation of inflammatory cells, thickening of the alveolar walls, and progressive disruption of normal alveolar architecture, collectively indicating exacerbated pulmonary inflammation and tissue injury [33,34]. Consistent with this mechanism, previous studies have demonstrated that TXNIP contributes to the regulation of Asian dust-induced pulmonary inflammation [13]. In the present study, AD treatment markedly attenuated Th1-associated cytokine production and reduced inflammatory cell infiltration, including the accumulation of immune cells in the alveoli and bronchi regions. Furthermore, AD suppressed activation of the TXNIP/NLRP3 inflammasome pathway and lowered the expression of caspase-1 and IL-1β in both PM-stimulated RAW264.7 cells and PM-exposed mice. These findings suggest that the protective effects of AD against PM-induced inflammation may be mediated, at least in part, through inhibition of TXNIP/NLRP3 inflammasome signaling. Treatment with AD and TXNIP-specific siRNA further enhanced these inhibitory effects, leading to greater suppression of NLRP3 activation and stronger inhibition of mitochondrial apoptotic signaling than treatment with AD alone. Activation of TXNIP/NLRP3 inflammasome signaling exacerbates inflammatory responses in lung inflammation models induced by exposure to dust and nanoparticles, consistent with our reported findings [13,35,36]. Therefore, pharmacological inhibition of TXNIP/NLRP3 inflammasome signaling by AD markedly attenuated PM-induced lung inflammation. Collectively, AD confers protective effects against lung inflammation both in vitro and in vivo through targeting the TXNIP-mediated inflammatory pathway.

The genus *Adenophora* has long been recognized in traditional Korean medicine for its diverse pharmacological properties, including anti-inflammatory, anti-allergic, and spleen protective effects [16,37,38]. However, the pharmacological activity of AD has not been fully characterized. Previous phytochemical and pharmacological studies identified saponins, flavonoids, and polysaccharides as the major bioactive constituents in AD [39,40]. In addition, *A. triphylla* var. japonica has been reported to attenuate inflammatory and allergic responses by reducing the release of pro-inflammatory cytokines and decreasing the expression of inducible nitric oxide synthase (iNOS) and cyclooxygenase-2 (COX-2) in ovalbumin (OVA)-challenged mouse models and LPS-stimulated RAW264.7 cells [18]. Moreover, oral treatment with *A. triphylla* var. japonica extract (100–400 mg/kg/day) for 11 days did not produce detectable systemic or organ toxicity in mice, which was supported by normal hematological parameters and the absence of abnormal histopathological profiles [41]. In this study, AD attenuated airway inflammation by modulating of the TXNIP/NLRP3 inflammasome signaling pathway. This mechanistic distinction suggests that AD exerts its protective effects by targeting upstream oxidative stress-inflammasome signaling, thereby providing a novel molecular basis for the anti-inflammatory activity of the *Adenophora* species. Although the AD extract shows potential as a therapeutic agent, its clinical application remains limited by factors, such as pharmacokinetics, bioavailability, and safety in humans, which were not addressed in the current study. Nevertheless, further studies employing TXNIP knockout mice and adeno-associated virus (AAV)-mediated TXNIP overexpression models are warranted to validate the causal role of TXNIP in vivo and elucidate the therapeutic potential and molecular mechanisms of AD in PM-induced lung injury.

## 5. Conclusions

To the best of our knowledge, this study is the first to demonstrate that AD effectively alleviates TXNIP-mediated airway inflammatory responses in both PM-stimulated RAW264.7 cells and PM-exposed mice. Treatment with AD markedly ameliorated lung inflammatory cell infiltration, pro-inflammatory cytokine production, accumulation of inflammatory cells, and alveolar wall thickening by modulating the TXNIP/NLRP3 inflammasome pathway. These protective effects are mediated by the suppression of TXNIP-dependent NLRP3 inflammasome signaling. Collectively, these findings suggest AD as a promising plant-derived candidate with preventive potential against PM-induced lung inflammation.

## Figures and Tables

**Table 1:** Tentative identification of major peak detected in negative-ion mode of AD.

No.	R_t_ (min)	Identification	Precursor Mass (*m*/*z*)	Library Score	Fragment Ions (*m*/*z*)
**1**	0.97	(-)-Quinic acid	191.0562	92.7	173.0461, 127.039393.0342, 85.0290
**2**	1.28	D-Pyroglutamic acid	128.0353	100	82.0289
**3**	2.78	Pantothenic acid	218.1034	94.5	146.0828, 116.0732, 88.0408, 71.0504
**4**	3.6	Neochlorogenic acid	353.0873	95.9	191.0554, 161.0247, 135.0450
**5**	5.04	Chlorogenic acid	353.0875	99.5	191.0538
**6**	5.42	trans-2-Hydroxycinnamic acid	163.0411	82.5	145.8940, 119.0511
**7**	5.43	Cryptochlorogenic acid	353.0888	95.8	191.0559, 173.0469, 135.0449, 93.0345
**8**	5.54	Herbacetin-3,8-diglucopyranoside	625.1408	99.2	625.1411, 462.0811, 299.0204
**9**	6.8	L-Malic acid	133.0152	98.3	115.0032, 72.9926
**10**	7.15	Luteolin-7,3′-di-O-glucoside	609.1452	100	447.0919, 285.0397
**11**	7.6	3-Methoxysalicylic acid	167.0358	93.6	167.0353, 108.0220
**12**	8.48	Rutin	609.1450	98.4	301.0351, 300.0260, 271.0250
**13**	8.77	Kaempferol-7-O-neohesperidoside	593.1500	99.1	285.038
**14**	8.88	Isoferulic acid	193.0514	86.3	178.0275, 134.0379, 133.0298
**15**	8.98	Luteolin-7-O-β-D-glucoside	447.0926	100	285.0375
**16**	9.13	Kaempferol-3-O-rutinoside	593.1504	97.1	285.0396
**17**	10.13	Apigenin 7-glucoside	431.0974	100	269.0446, 268.0351
**18**	10.33	Neodiosmin	607.1659	100	299.0531, 284.0308
**19**	10.42	Azelaic acid	187.0970	99.6	169.0879, 125.0962, 123.0813, 97.0655
**20**	10.71	Luteolin-4′-O-glucoside	447.0940	99.9	285.0401
**21**	12.46	Luteolin	285.0398	99.3	151.0038, 133.0289, 107.0138
**22**	14.05	Apigenin	269.0450	95.1	151.0040, 117.0347, 107.0138
**23**	14.05	Undecanedioic acid	215.1283	99.5	197.1175, 153.1284
**24**	14.71	13S-Hydroxy-9Z,11E,15Z-octadecatrienoic acid	293.2121	96.0	275.2020, 223.1341, 195.1390
**25**	25.01	9(10)-Epoxy-12Z-octadecenoic acid	295.2269	96.3	277.2150, 171.1015
**26**	26.86	13-Keto-9Z,11E-octadecadienoic acid	293.2113	99.6	249.2232, 133.0966
**27**	27.21	Betulinic acid	455.3528	100	455.3523
**28**	29.12	Alpha-Linolenic acid	277.2179	100	277.216
**29**	29.14	Linoleic acid	279.2325	100	279.2313
**30**	29.79	Oleic acid	281.2491	100	281.2482

**Table 2:** Tentative identification of major peak detected in positive-ion mode of AD.

No.	R_t_ (min)	Identification	Precursor Mass (*m*/*z*)	Library Score	Fragment Ions (*m*/*z*)
**1**	0.95	Betaine	118.0865	100	59.0729, 58.0647
**2**	1.26	D-Pyroglutamic acid	130.0504	80.0	84.0444,56.0497
**3**	1.5	Isoleucine	132.1028	99.2	86.0975, 69.0710
**4**	5.05	Chlorogenic acid	355.1018	98.7	163.0380, 145.0291, 117.0345
**5**	6.5	Norharmane	169.0770	98.6	115.0552
**6**	8.96	Astragalin	449.1071	100	287.0525
**7**	9.11	Datiscin	595.1663	97.8	449.1067, 287.0546, 85.0295
**8**	21.2	Ethyl 4-hydroxybenzoate	167.0356	94.3	139.0403, 121.0296, 95.0498, 77.0384
**9**	24.95	Monolinolenin (9c,12c,15c)	353.2700	97.7	335.2647, 261.2288, 243.2124
**10**	25.87	9,12-Octadecadiynoic acid	277.2164	94.1	259.2061, 149.1343, 135.1181
**11**	26.45	1-Pentadecanoyl-sn-glycero-3 phosphocholine	482.3265	92.0	299.2597, 184.0747, 104.1084
**12**	26.56	1-Monolinoleoyl-rac-glycerol	355.2857	96.3	337.2775, 263.2380, 95.0868, 81.0710
**13**	26.78	Glycyrrhetinic acid	471.3486	86.3	425.3436, 235.1714, 217.1607, 189.1654
**14**	27.2	13-Keto-9Z,11E-octadecadienoic acid	295.2266	86.5	277.2167, 179.1435
**15**	27.49	1-Palmitoyl-sn-glycero-3-phosphocholine	496.3399	96.0	478.3289, 184.0724, 104.1070
**16**	29.14	Stearidonic acid	277.2183	83.7	163.1164, 149.1358, 121.1030
**17**	29.69	Oleamide	282.2777	92.8	265.2522, 247.2414, 149.1329, 135.1171, 95.0857, 69.0692
**18**	30.08	Monoolein	357.3019	87.5	339.2909, 265.2554, 247.2437, 135.1187, 95.0865
**19**	30.45	Tridemorph	298.3122	91.6	116.1106, 102.0922, 57.0710
**20**	30.74	Lupenone	425.3776	89.4	407.3662, 187.1488, 161.1334, 95.0864
**21**	32.28	Cinnamic acid	149.0247	100	121.0299, 93.0346, 65.0393
**22**	32.74	Erucamide	338.3407	94.1	321.3148, 303.3044, 163.1490, 135.1177, 97.1017
**23**	33.67	Campesterol	383.3678	86.7	257.2291, 201.1669, 161.1339, 95.0867, 81.0715

## Data Availability

The original contributions presented in this study are included in the article. Further inquiries can be directed to the corresponding authors.

## References

[1] Liu H., Zhang X., Sun Z., Chen Y. (2023). Ambient Fine Particulate Matter and Cancer: Current Evidence and Future Perspectives. Chem. Res. Toxicol..

[2] Hashizume M., Kim Y., Ng C.F.S., Chung Y., Madaniyazi L., Bell M.L., Guo Y.L., Kan H., Honda Y., Yi S.M. (2020). Health Effects of Asian Dust a systematic review and meta-analysis. Environ. Health Perspect..

[3] Yu W., Ye T., Zhang Y., Xu T., Lei Y., Chen Z., Yang Z., Zhang Y., Song J., Yue X. (2023). Global estimates of daily ambient fine particulate matter concentrations and unequal spatiotemporal distribution of population exposure: A machine learning modelling study. Lancet Planet. Health.

[4] Aryal A., Harmon A.C., Dugas T.R. (2021). Particulate matter air pollutants and cardiovascular disease: Strategies for intervention. Pharmacol. Ther..

[5] Van Eeden S.F., Tan W.C., Mukae H., Terashima T., Fujii T., Qui D., Vincent R., Hogg J.C. (2001). Cytokines involved in the systemic inflammatory response induced by exposure to particulate matter air pollutants (PM(10)). Am. J. Respir. Crit. Care Med..

[6] Kim H.J., Song J.Y., Park T.I., Choi W.S., Kim J.H., Kwon O.S., Lee J.Y. (2022). The effects of BRL-50481 on ovalbumin-induced asthmatic lung inflammation exacerbated by co-exposure to Asian sand dust in the murine model. Arch. Pharmacal Res..

[7] Chen Q., Wang M., Sun H., Wang X., Wang Y., Li Y., Zhang L., Mu Z. (2018). Enhanced health risks from exposure to environmentally persistent free radicals and the oxidative stress of PM_2.5_ from Asian dust storms in Erenhot, Zhangbei and Jinan, China. Environ. Int..

[8] Han X., Wu Y.C., Meng M., Sun Q.S., Gao S.M., Sun H. (2019). Linarin prevents LPS-induced acute lung injury by suppressing oxidative stress and inflammation via inhibition of TXNIP/NLRP3 and NF-κB pathways. Int. J. Mol. Med..

[9] Zhou R., Tardivel A., Thorens B., Choi I., Tschopp J. (2010). Thioredoxin-interacting protein links oxidative stress to inflammasome activation. Nat. Immunol..

[10] Wu J., Yan Z., Schwartz D.E., Yu J., Malik A.B., Hu G. (2013). Activation of NLRP3 inflammasome in alveolar macrophages contributes to mechanical stretch-induced lung inflammation and injury. J. Immunol..

[11] Dostert C., Petrilli V., Van Bruggen R., Steele C., Mossman B.T., Tschopp J. (2008). Innate immune activation through Nalp3 inflammasome sensing of asbestos and silica. Science.

[12] Zheng R., Tao L., Jian H., Chang Y., Feng Y., Zhang H. (2018). NLRP3 inflammasome activation and lung fibrosis caused by airborne fine particulate matter. Ecotoxicol. Environ. Saf..

[13] Pak S.W., Kim W.I., Lee S.J., Park S.H., Cho Y.K., Kim J.S., Kim J.C., Kim S.H., Shin I.S. (2024). TXNIP regulates pulmonary inflammation induced by Asian sand dust. Redox Biol..

[14] Cheon K.S., Kim K.A., Yoo K.O. (2017). The complete chloroplast genome sequences of three *Adenophora* species and comparative analysis with Campanuloid species (Campanulaceae). PLoS ONE.

[15] Ham Y.A., Choi H.J., Kim S.H., Chung M.J., Ham S.S. (2009). Antimutagenic and antitumor effects of *Adenophora triphylla* extracts. J. Korean Soc. Food Sci. Nutr..

[16] Choi H.J., Kim S.H., Oh H.T., Chung M.J., Cui C.B., Ham S.S. (2008). Effects of *Adenophora triphylla* ethylacetate extract on mRNA levels of antioxidant enzymes in human HepG2 cells. J. Korean Soc. Food Sci. Nutr..

[17] Lee M.Y., Seo C.S., Lee J.A., Lee N.H., Kim J.H., Ha H., Zheng M.S., Son J.K., Shin H.K. (2011). Anti-asthmatic effects of *Angelica dahurica* against ovalbumin-induced airway inflammation via upregulation of heme oxygenase-1. Food. Chem. Toxicol..

[18] Jang H.H., Kim M.J., Cho S.Y., Kim J.B., Lee S.H., Lee Y.M. (2015). Anti-Inflammatory and Anti-Allergic Effects of *Adenophora triphylla* var. japonica Extract. J. East Asian Soc. Diet. Life.

[19] Lee J.E., Jang K.H., Lee D.H. (2010). Antioxidant, Anti-inflammatory and Anticancer Activities by Different Parts in Codonopsis pilosuala (FR.) NANNF. and *Adenophora triphylla* var. japonica Hara. J. Korean Soc. Food Sci. Nutr..

[20] Saggini R., Pellegrino R. (2024). MAPK is implicated in sepsis, immunity, and inflammation. Int. J. Infect..

[21] Chung K.J., Jung H.J., Jung S.K., Rhee H.K. (2002). The inhibitory effects of Sabaek-san and Sabaeksan plus Sasam on the IL-6, IL-8 and GM-CSF mRNA levels in human epithelial cells. J. Korean Soc. Food Sci. Nutr..

[22] Ha J.H., Lee B.W., Yi D.H., Lee S.J., Kim W.I., Pak S.W., Kim H.Y., Kim S.H., Shin I.S., Kim J.C. (2024). Particulate matter-mediated oxidative stress induces airway inflammation and pulmonary dysfunction through TXNIP/NF-κB and modulation of the SIRT1-mediated p53 and TGF-β/Smad3 pathways in mice. Food Chem. Toxicol..

[23] Son J.Y., Kwack W.G., Chung E.K., Shin S., Choi Y.J. (2022). Effects of early initiation of high-dose dexamethasone therapy on pro-inflammatory cytokines and mortality in LPS-challenged mice. Healthcare.

[24] Lee B.W., Ha J.H., Yi D.H., Kim J.H., Jeong S.H., Lee H.J., Kim Y.H., Kwon H.J., Park J.Y., Kim W.S. (2025). *Spiraea prunifolia* var. *simpliciflora* leaves ameliorate inflammatory responses and oxidative stress in PPE/LPS-induced chronic obstructive pulmonary disease mouse model. Front. Pharmacol..

[25] Lee B.W., Ha J.H., Shin H.G., Jeong S.H., Kim J.H., Lee J., Park J.Y., Kwon H.J., Jung K.S., Lee W.S. (2020). *Lindera Obtusiloba* Attenuates Oxidative Stress and Airway Inflammation in a Murine Model of Ovalbumin-Challenged Asthma. Antioxidants.

[26] Kim K.H., Kabir E., Kabir S. (2015). A review on the human health impact of airborne particulate matter. Environ. Int..

[27] Valavanidis A., Fiotakis K., Valchogianni T. (2008). Airborne particulate matter and human health: Toxicological assessment and importance of size and composition of particles for oxidative damage and carcinogenic mechanisms. J. Environ. Sci. Health C Environ. Carcinog. Ecotoxicol. Rev..

[28] Pan M., Zhang F., Qu K., Liu C., Zhang J. (2022). TXNIP: A double-edged sword in disease and therapeutic outlook. Oxidative Med. Cell. Longev..

[29] Li J., An Z., Song J., Du J., Zhang L., Jiang J., Ma Y., Wang C., Zhang J., Wu W. (2021). Fine particulate matter-induced lung inflammation is mediated by pyroptosis in mice. Ecotoxicol. Environ. Saf..

[30] Meng M. (2017). Digitoflavone (DG) attenuates LPS-induced acute lung injury through reducing oxidative stress and inflammatory response dependent on the suppression of TXNIP/NLRP3 and NF-κB. Biomed. Pharmacother..

[31] Lee B.W., Ha J.H., Yi D.H., Kim J.H., Jeong S.H., Jeong J.H., Jung K., Jeong H.J., Park J.Y., Kim W.S. (2026). *Boehmeria nivea* (L.) Gaud. Ameliorates Oxidative stress-mediated Inflammatory Responses and Apoptosis in LPS/CSC-induced Chronic Obstructive Pulmonary Disease Mouse Model. J. Ethnopharmacol..

[32] Wang S., Hu L., Fu Y., Xu F., Shen Y., Liu H., Zhu L. (2024). Inhibition of IRE1α/XBP1 axis alleviates LPS-induced acute lung injury by suppressing TXNIP/NLRP3 inflammasome activation and ERK/p65 signaling pathway. Respir. Res..

[33] Lam M., Barry K.T., Hodges C.J., Harpur C.M., Ong J.D.H., Rosli S., West A.C., Dousha L., Hertzog P.J., Mansell A. (2025). NLRP3 deficiency abrogates silica-induced neutrophil infiltration, pulmonary damage and fibrosis. Respir. Res..

[34] Paik S., Kim J.K., Shin H.J., Park E.J., Kim I.S., Jo E.K. (2025). Updated insights into the molecular networks for NLRP3 inflammasome activation. Cell. Mol. Immunol..

[35] Kim W.I., Pak S.W., Lee S.J., Park S.H., Lim J.O., Kim D.I., Shin I.S., Kim S.H., Kim J.C. (2024). Copper oxide nanoparticles exacerbate chronic obstructive pulmonary disease by activating the TXNIP-NLRP3 signaling pathway. Part. Fibre Toxicol..

[36] Kim W.I., Pak S.W., Lee S.J., Park S.H., Lim J.O., Shin I.S., Kim J.C., Kim S.H. (2024). Copper Oxide Nanoparticles Induce Pulmonary Inflammation and Exacerbate Asthma via the TXNIP Signaling Pathway. Int. J. Mol. Sci..

[37] Park S.M., Jung C.J., Lee D.G., Choi B.R., Ku T.H., La I.J., Cho I.J., Ku S.K. (2022). *Adenophora Stricta* Root Extract Protects Lung Injury from Exposure to Particulate Matter _2.5_ in Mice. Antioxidants.

[38] Sun W., Zeng C., Yue D., Liu S., Ren Z., Zuo Z., Deng J., Peng G., Hu Y. (2019). *Ageratina adenophora* causes spleen toxicity by inducing oxidative stress and pyroptosis in mice. R. Soc. Open Sci..

[39] Lee H.H., Kim H.J., Yeon S.W., Ryu S.H., Turk A., Hwang B.Y., Lee M.K. (2026). Phenylpropanoids and polyacetylenes from *Adenophora triphylla*. Nat. Prod. Res..

[40] Chun J., Kang M., Kim Y.S. (2014). A triterpenoid saponin from *Adenophora triphylla* var. japonica suppresses the growth of human gastric cancer cells via regulation of apoptosis and autophagy. Tumour Biol..

[41] Hu J.R., Jung C.J., Ku S.M., Jung D.H., Ku S.K., Choi J.S. (2019). Antitussive, expectorant, and anti-inflammatory effects of *Adenophora* Radix powder in ICR mice. J. Ethnopharmacol..

